# Chemical incompatibility between formation and injection water: implications for oil recovery in porous media

**DOI:** 10.3389/fchem.2025.1621714

**Published:** 2025-06-18

**Authors:** Jiangtao Wang, Xiaolong Wan, Junsong Ren, Genggeng Zhu, Wei Xu, Yingxue Hu

**Affiliations:** ^1^ School of Human Settlements and Civil Engineering, Xi’an Jiaotong University, Xi’an, China; ^2^ Oil Production Plant NO.5 of Changqing Oilfield, China National Petroleum Corporation, Xi’an, China; ^3^ Exploration and Development Institute, PetroChina Changqing Oilfield Company, Xi’an, China; ^4^ Foreign Cooperation Department, PetroChina Changqing Oilfield Company, Xi’an, China

**Keywords:** chemical incompatibility, formation-injection water interaction, scale deposition, colloidal dynamics, surfactant flooding

## Abstract

Low-salinity water flooding is widely recognized as an effective enhanced oil recovery (EOR) method, primarily by altering wettability and reducing interfacial tension. However, chemical incompatibility between injected water and formation water may induce scale deposition, leading to pore blockage and injectivity impairment, thereby posing significant challenges to EOR efficiency. A better understanding of the interplay between chemical incompatibility and pore-scale oil-water interface dynamics is crucial for optimizing waterflooding performance, particularly in low-permeability reservoirs. This study integrates ion characterization, colloidal analysis, solubility product calculations, and microfluidic visualization to systematically evaluate the compatibility of formation and injected waters, while directly observing pore-scale fluid displacement processes. Results reveal that ionic composition analysis reveals significant incompatibility between the sulfate-rich injection water and calcium/barium-containing formation water, creating conditions favorable for mineral scaling. Subsequent examination of scaling dynamics demonstrates that incompatible fluid mixing initiates nanoparticle formation, which progresses through two distinct growth pathways: coalescence-driven crystal enlargement and aggregation-dominated cluster formation, ultimately leading to pore-throat obstruction. Microfluidic visualization shows residual oil persists primarily as interfacial films and pore-center clusters after initial waterflooding, with their spatial arrangement governed by salinity-dependent wettability alteration and capillary forces. The introduction of incompatible water further exacerbates fluid trapping through capillary valve effects—a capillary-driven resistance occurring when interfacial forces oppose fluid advancement at pore-throat junctions—creating stagnant zones that promote particle accumulation. Pressure monitoring during flooding experiments reveals characteristic response patterns: an initial pressure peak during waterflooding, followed by secondary pressure elevation due to scale deposition, and subsequent partial pressure reduction through surfactant-mediated interfacial tension reduction and wettability modification. A self-reinforcing cycle emerges, coupling ion incompatibility, capillary trapping, and precipitate growth, encapsulated in a colloid-capillary coupling framework. To disrupt this cycle, a synergistic chemical strategy combining surfactants and scale inhibitors is proposed, simultaneously enabling interface modification and nucleation suppression to enhance sweep efficiency. This integrated approach provides a mechanistic foundation for optimizing waterflooding in chemically complex reservoirs, achieving a balanced synergy between interfacial control and scale mitigation.

## 1 Introduction

Water flooding has long been recognized as one of the most widely used and cost-effective secondary oil recovery techniques, particularly in mature oilfields where primary recovery methods are no longer sufficient to maintain production levels (Arab et al., 2020; Mouret et al., 2023; Gong et al., 2024). By injecting water into the reservoir, the remaining oil is mobilized and displaced toward production wells, leading to enhanced recovery rates. However, despite its widespread application, water flooding is often accompanied by a number of challenges (Ghasemian et al., 2019; Razavirad et al., 2024), especially when there is a chemical mismatch between the injected water and the native formation water (Tohidi and Sadeghnejad, 2021; Bijani et al., 2023; Valadbeygian et al., 2023). This mismatch can lead to a cascade of geochemical reactions, resulting in adverse effects such as scaling, fines migration, and pore throat blockage—phenomena that significantly impair reservoir permeability and productivity (Khormali et al., 2018; Shojaee et al., 2023; Zakian and Moghadas, 2025).

One of the primary concerns in water injection processes is the chemical incompatibility between the injected and formation waters (Stalker et al., 2003; Rostami et al., 2019; Hosseini et al., 2023). Injection waters derived from surface sources, desalinated seawater (Shojaee et al., 2023), or treated industrial brines frequently differ in ionic composition from formation brines that have been in chemical equilibrium with the reservoir rock for geological time scales (Golghanddashti et al., 2013; Kluk, 2020). For instance, the introduction of sulfate-rich injection water into carbonate- or sandstone-based reservoirs rich in calcium and barium can lead to the precipitation of sparingly soluble salts such as barite (BaSO_4_) (Valadbeygian et al., 2023) or gypsum (CaSO_4_) (Rosa et al., 2015; Abib et al., 2018; Mahmoodi and Nick, 2022; Pramana et al., 2023). Similarly, the dilution of divalent cation-rich formation waters with low-salinity injection water can disturb the ion exchange balance, causing clay minerals to swell or migrate, leading to fines plugging and mechanical damage to pore structures (Mohsenzadeh et al., 2015; Mohammadi and Riahi, 2020).

In sandstone reservoirs, where reactive minerals such as feldspars, smectite, illite, and chlorite are present, water-rock interactions are particularly complex. Reactions including ion exchange, mineral dissolution, and secondary precipitation may alter the reservoir’s pore structure, flow capacity, and wettability (Mohammadi and Riahi, 2020). While many studies have employed core flooding, zeta potential analysis, and scaling prediction models to evaluate these processes, (Fu et al., 2012; Haghtalab et al., 2015; Ahmadi et al., 2017; Tohidi and Sadeghnejad, 2021; Mahmoodi and Nick, 2022; Zojaji et al., 2022) such approaches often offer only macroscopic insights. They typically fail to capture the real-time, pore-scale evolution of chemical and physical changes induced by incompatible water injection. This lack of spatial resolution obscures our understanding of how and where blockage initiates and progresses within the reservoir rock, making it difficult to devise effective mitigation strategies.

Moreover, conventional monitoring techniques such as effluent analysis, pressure monitoring, and porosity-permeability correlations provide indirect evidence of formation damage, but they are unable to visualize fluid-fluid and fluid-solid interactions at the pore scale. With the advancement of visualization tools such as microfluidic chips, X-ray microtomography, and real-time optical imaging, it is now possible to directly observe the migration and transformation of particles and precipitates in porous media. Microfluidic models, in particular, have emerged as powerful experimental platforms that mimic the geometry and complexity of pore networks under controlled conditions, allowing researchers to investigate the dynamic behavior of colloidal particles, scaling species, and emulsions during water injection.

While recent studies (Shojaee et al., 2023; Gong et al., 2024) have examined water compatibility in various reservoirs, their bulk- and core-scale approaches lack real-time resolution at the pore scale. Amiri et al. (2025) further explored this issue using microfluidic visualization to investigate pore-scale scale formation under incompatible injection conditions, demonstrating the utility of NaOH pretreatment for reducing blockage formation. Their work underscores the need for real-time, geometry-resolved analysis to fully understand chemical damage mechanisms. Our work extends this line of research by integrating microfluidic visualization, colloidal analysis, and dynamic pressure monitoring, thereby providing mechanistic insights into capillary-blockage coupling effects under chemically incompatible conditions.

Despite these technological advances, there remains a significant gap in understanding how precisely the incompatibility between formation and injection water translates into pore-scale blockage and flow impairment. In particular, the interplay between particle generation, aggregation, and transport in the context of oil-water displacement remains poorly resolved. To bridge that gap, this study employs microfluidic visualization in a transparent chip etched with a two-dimensional porous network, enabling real-time observation of flow behavior, particle formation, and blockage under different water chemistry conditions. By replicating the injection of ion-incompatible water into the chip, we visualize the formation and development of chemically induced blockage.

This work provides a mechanistic foundation for optimizing water injection strategies and addresses a central research question: How does water chemistry mismatch influence pore-scale displacement behavior and mineral scaling in low-permeability reservoirs? Answering this question is essential for developing effective strategies to mitigate formation damage and improve oil recovery efficiency.

## 2 Materials and methods

### 2.1 Fluids

The transition zone between the secondary injection water from the Luohe Formation (injection water) and the primary injection water from the Chang 3 Member (formation water) is influenced not only by capillary forces in the pore throats but also by the chemical composition of the two water bodies. As evident from the results of physical simulation experiments, the compatibility between the two is poor. To determine the compatibility between the two formation waters, we collected water samples from the injection water and the formation water to conduct macro-scale compatibility experiments.

The two water sources were mixed in mass ratios of injection water to formation water of 3:1, 2:1, 1:1, 1:2, and 1:3 (Table 1). After sealing, the mixtures were kept at a constant temperature of 65°C for 144 h, and the final state of the mixed water samples was obtained. Under different mixing ratios, the injection water and formation water easily produced powdery and granular precipitates. To identify these precipitates, chromatographic analysis was performed on the water samples. The results of ion chromatographic analysis showed significant differences in Ca^2+^, Ba^2+^, and SO_4_^2-^ between the two formation waters (Table 2). The Ca^2+^ content in the formation water ranged from 3272.34 to 4584.14 mg/L, while it was only 47.14–85.1 mg/L in the injection water. The Ba^2+^ content in the formation water was between 211.32 and 297.3 mg/L, compared to only 2.18–3.44 mg/L in the injection water. The SO_4_^2-^ content was 72.38 mg/L in the formation water and 1447.48 mg/L in the injection water. Therefore, when the two formation waters are mixed, they are prone to produce CaSO_4_ and BaSO_4_ precipitates.

**TABLE 1:** Precipitation after mixing injection water and formation water.

Ratio (v/v)	3:1	2:1	1:1	1:2	1:3
Colour tone	Nearly colourless, very faint pale yellow	Almost colourless with a trace of yellow	Slight yellow tint, still largely colourless	Light yellow	Distinct pale-to-light yellow

**TABLE 2:** Ion chromatography analysis of water samples.

Ion types (mg/L)	2021.08.2		2022.07.22	
	Formation water	Injection water	Formation water	Injection water
Na^+^	58063.88	982.44	30321	530.62
K^+^	435.06	3.98	263.14	2.6
Ca^2+^	4584.14	85.1	3272.34	47.14
Mg^2+^	829.48	33.78	395.54	27.36
Sr^2+^	256.1	0.64	183.68	1.24
Ba^2+^	297.3	3.44	211.32	2.18
F^−^	15.28	27.16	7.3	1.04
Cl^−^	102233.58	309.28	57753.52	237
Br^−^	95.74	64.94	78.62	0
NO_3_^−^	67.76	0	69.12	59.38
SO_4_^2-^	72.38	1447.48	447.14	752.36
HCO_3_^−^	182.65	204.59	353.8	366
CO_3_^2-^	325.29	156.95	0	0
pH	6.8	6.8	6.9	6.8
Salinity	167458.64	3319.78	93356.52	2026.92

### 2.2 Porous media

The test sample was taken from low-permeability reservoirs. To obtain the chip pore channels, we used 2D channel slices based on CT scanning and reconstructed the pore network numerically, followed by wet etching to create the required chip. The specific production process is as follows:

(1) Sample preparation: Select the typical core of low-permeability reservoirs, wash oil, dry and drill string.
(2) CT scanning: Use X-ray CT equipment to perform core slicing with a resolution of no less than 0.8 μm and ensure that the field width is not less than 2 cm. Conduct more than 1000 scans to ensure a nearly complete reconstruction of the entire core, ensuringthat the selected 2D slices are representative.
(3) Selection of Typical Slices: Based on the obtained real 2D network channels, select slices that can represent the basic geological characteristics of the oilfield core, such as porosity, permeability, average pore diameter, connectivity, and coordination number. These characteristics should be at an average level, and each pore should have a certain degree of connectivity to avoid dead pores.
(4) Image Binarization: Set the grayscale value of the pixels in the image to either 0 or 255, creating a clear visual effect of only black and white.
(5) Image Erosion and Dilation: First, perform erosion, which involves moving the structural element such that if the intersection with the original image completely belongs to the original image area, that position is preserved. All points meeting this condition form the erosion result of the original image by the structural element. Then, perform dilation, which involves convolving the structural element over the original image. If there is an overlap between the structural element and the original image during movement, record that position. The collection of all intersecting positions constitutes the dilation result under the structural element.
(6) Pore network vectorization: Based on the selected CT channels, output the reconstructed pore network, vectorize it, and proceed with chip fabrication.

### 2.3 Experimental setup

**FIGURE 1:**
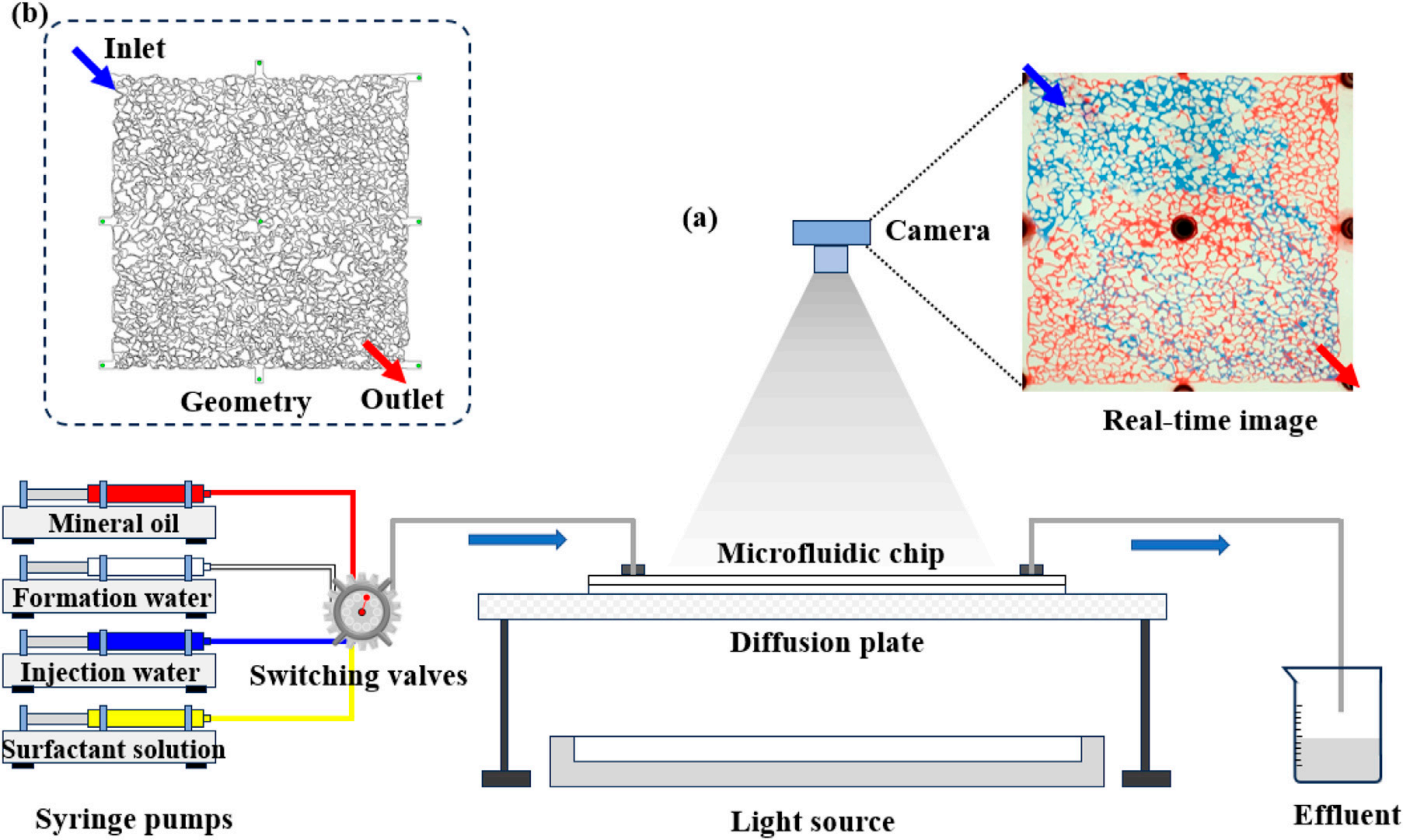
Experimental procedure schematic: **(a)** schematic diagram of the experimental apparatus; **(b)** microfluidic chip design.

Based on the pore network model obtained from the above CT scan images, the CAD diagram of the large size reservoir chip is designed and the glass base large size reservoir chip is processed (Figure 1a).

(1) Etching: Based on the two-dimensional pore distribution map, a corresponding mask is created. The mask is then placed over the glass, and acid is poured over it. The acid penetrates through the pores in the mask, etching channels to a depth of 100 μm.
(2) Firing: Another piece of glass is bonded to the etched glass, dried, and the chip is completed. Additionally, small holes with a diameter of 1 mm are drilled at the four corners of the chip for fluid injection.
(3) Seal tightness test: The large-scale microfluidic chip is placed horizontally in the center of a specialized test platform. The German Cetoni microfluidic precision syringe pump (Figure 1) is connected to the corresponding inlet to test the chip’s seal tightness. The Cetoni microfluidic precision syringe pump (constant flow pump) has a flow control range of 1.3 nL–300 mL/min, can measure pressure, and operates without pressure oscillations.
(4) Testing and photography: Depending on the experimental objectives, the liquid injection sequence and flow rate are adjusted. An ultra-resolution camera matrix is used for continuous image acquisition of the displacement process (Figure 1b) for subsequent data analysis.
(5) Injection Protocol: The experiment consisted of three consecutive injection stages, all performed at a constant flow rate of 2 μL/min. First, formation water was injected until breakthrough was observed at the outlet. This was followed by injection water, also injected until a new steady flow was reached, indicating completion of the second stage. Finally, a surfactant solution composed of 3% COA-2G and 0.5% NH_4_Cl (dissolved in injection water) was introduced and continued until no additional oil was observed at the outlet. This breakthrough-based injection strategy ensured that each stage realistically reflected flow behavior under displacement and scale formation conditions.

## 3 Results and discussion

### 3.1 Water compatibility test

**FIGURE 2:**
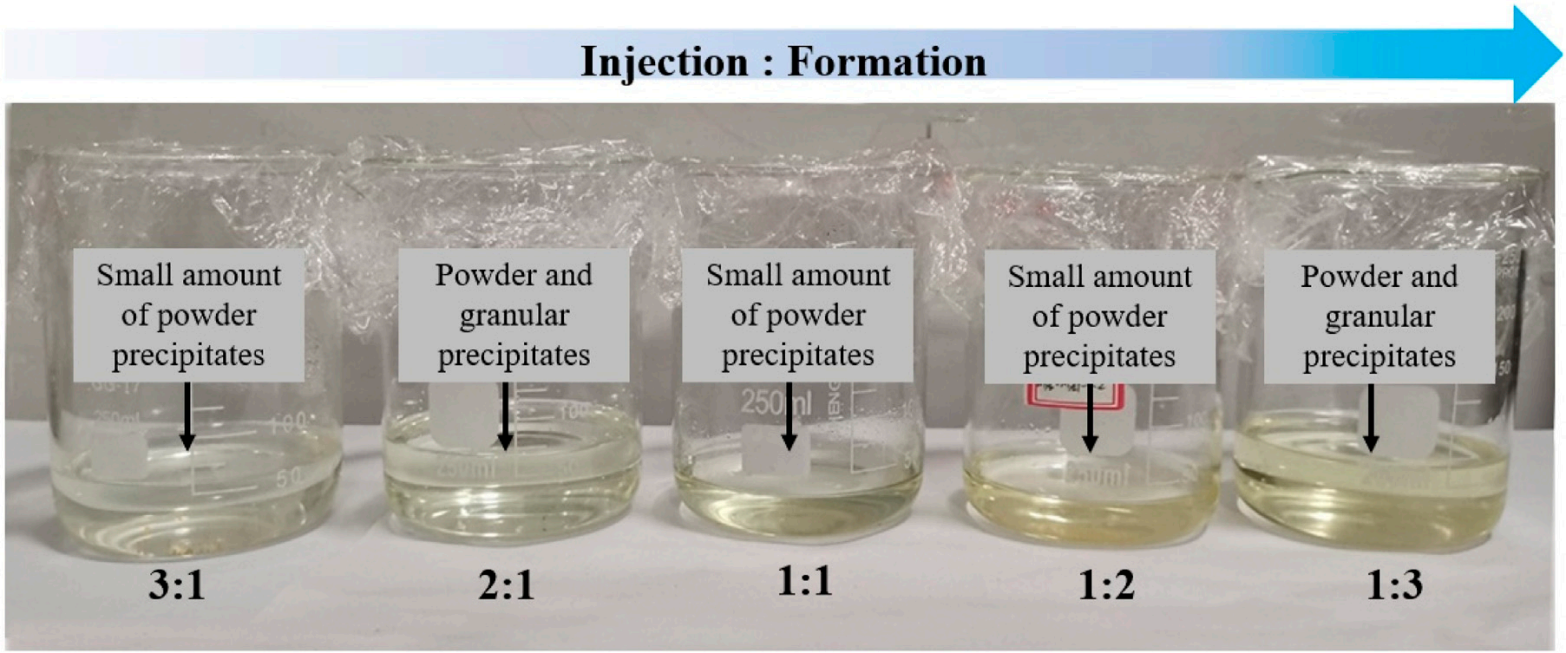
Initial appearance of mixed water samples at various injection water to formation water ratios.

To preliminarily assess the compatibility between the injection water and formation water, the two were mixed at five different mass ratios: 3 : 1, 2 : 1, 1 : 1, 1 : 2, and 1 : 3. Figure 2 displays the five glass beakers (250 mL) containing these mixtures, arranged from left to right in order of decreasing injection-water fraction. Every beaker holds a completely clear, colourless–to–very-pale-yellow solution, and no particles, haze, or settling are visible—confirming that no precipitate has formed at the moment of blending and that all mixtures are initially homogeneous.

**FIGURE 3:**
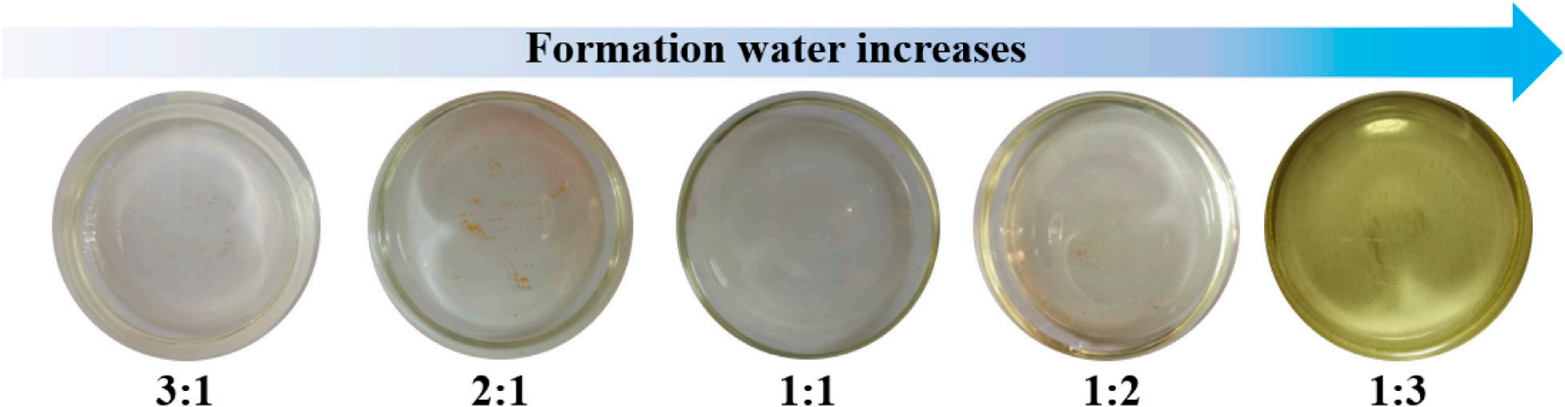
Observation of sample reactions at different injection water to formation water ratios. (After standing at 65°C for 144 h).

After sealing, the samples were placed in a constant-temperature oven at 65°C for 144 h to simulate subsurface conditions. As shown in Figure 3, significant differences in the visual appearance of the samples emerged over time. Some mixtures exhibited varying degrees of turbidity or precipitate formation, suggesting ion exchange reactions or scaling phenomena due to poor compatibility at certain mixing ratios. These visual changes provide preliminary but direct evidence of potential scaling risks and formation damage that may occur during water injection operations.

**FIGURE 4:**
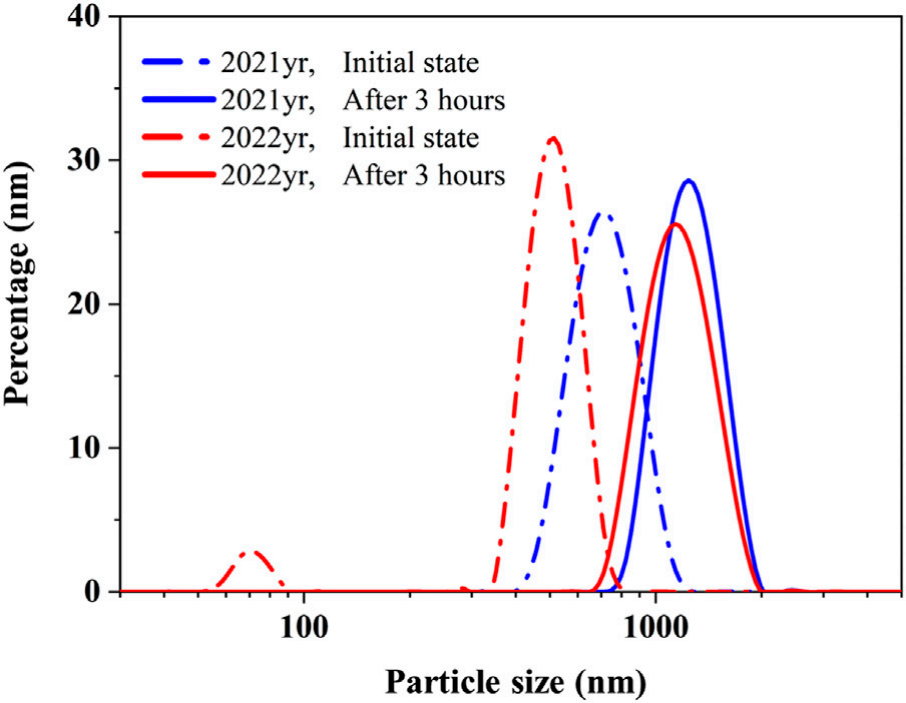
Particle size distribution at initial state and after 3 h in 2021 and 2022, measured by DLS.

Due to the high concentration of calcium ions (Ca^2+^) in the formation water and the elevated sulfate ion (SO_4_^2-^) concentration in the injection water, mixing these two water sources readily induces the formation of precipitates or colloidal particles through ionic reactions. To simulate this process, two sets of synthetic mixing solutions were prepared based on the measured ion concentrations from field samples collected on August 2, 2021, and July 22, 2022, respectively. Each solution reproduced the actual Ca^2+^ concentration in the formation water and SO_4_^2-^ concentration in the injection water at the time of sampling. The mixed solutions were kept at 65 °C for 3 h to simulate near-wellbore temperature conditions. After the reaction, a laser particle size analyzer was used to characterize the size distribution of the nanoparticles or colloids suspended in the solution. The measurement results are shown in Figure 4.

As shown in Figure 4, the particle size distribution of the solution prepared using the 2021 ion concentrations exhibited an overall shift toward larger sizes over time. Both the average particle size and the proportion of large particles increased, indicating continuous particle formation and aggregation within the solution. This suggests that under the 2021 conditions, the generation of new particles and their subsequent growth play a dominant role in the system’s behavior. In contrast, the solution prepared using the 2022 ion concentrations also showed an increase in average particle size over time; however, the proportion of large particles decreased. This implies that while particle aggregation occurred rapidly, the formation rate of new colloidal particles was relatively slower. As a result, the overall number of particles decreased as they coalesced into fewer, larger aggregates.

In both cases, calcium ions (Ca^2+^) reacted with sulfate ions (SO_4_^2-^) to first form nanoscale nuclei, which subsequently developed into colloidal substances. With extended time, these colloids aggregated and eventually settled as crystalline precipitates. The particle size distribution curves further confirm this behavior: In the 2021 sample solution, the distribution peak shifted rightward and increased in intensity, indicating active nucleation and crystal growth. In the 2022 sample, although the peak also shifted rightward, the peak intensity decreased, suggesting that aggregation of existing particles was the dominant mechanism. Both crystal growth and aggregation contribute to increasing particle size. Once the particles exceed a critical settling diameter, they begin to precipitate out of solution, potentially contributing to pore blockage in subsurface formations.

**FIGURE 5:**
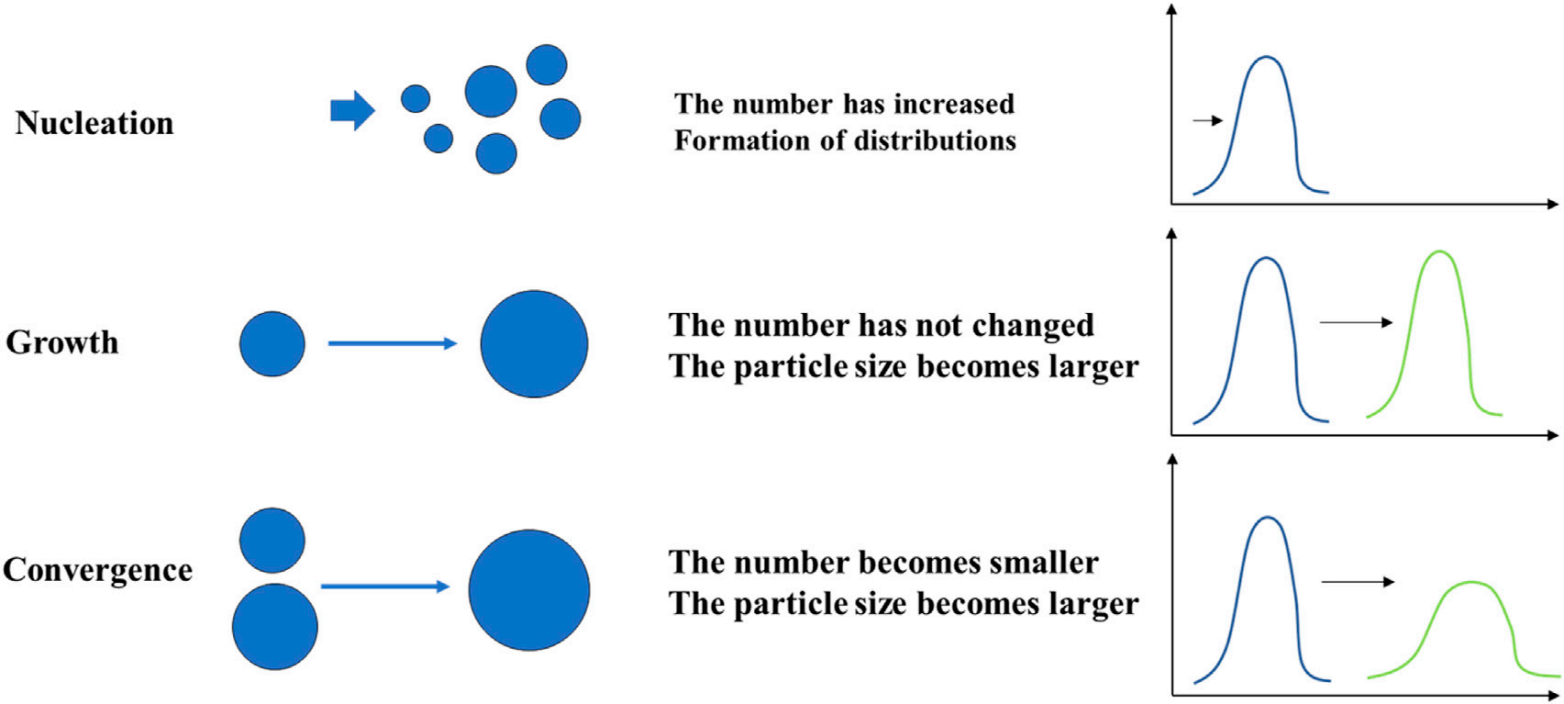
Schematic illustration of particle evolution from colloidal state to crystalline precipitate.

Figure 5 provides a schematic illustration of the evolution of particles formed in the mixed solution of injection water and formation water, where Ca^2+^ and SO_4_^2-^ react to produce insoluble species. The diagram captures the transformation process from initial nucleation of nanoparticles to colloidal aggregation, and eventually to the formation of crystalline precipitates. At the top of the figure, the system starts with the generation of dispersed primary nanoparticles due to supersaturation. These nanoparticles undergo two primary pathways: Path I: Coalescence Growth–Particles merge into larger structures through molecular-level diffusion and restructuring, resulting in increased particle size and a particle size distribution (PSD) that shifts toward larger sizes with intensified peaks. Path II: Aggregation-Dominated Growth–Existing particles aggregate without significant new nucleation, forming larger but fewer structures. The corresponding PSD exhibits a peak shift to the right, while the peak intensity decreases.

### 3.2 Scale deposition in throats

Based on the formation mechanism of stagnation zones in pore throats and water compatibility analysis mentioned above, the micro-mechanism of high-pressure injection deficiency is as follows: (1) At the intersection of throats and pores, capillary force changes from a driving force to a resistance, causing the oil-water interface movement to stop; (2) The stagnation zone within the throat provides a site for the mixing of two fluids; (3) Calcium sulfate/barium sulfate colloids formed within the throat hinder the inward diffusion of injection water; (4) Calcium sulfate/barium sulfate crystal particles continuously grow in this static region, ultimately blocking the throat, preventing injection water from passing through, reducing waterflood sweep efficiency, and increasing injection pressure.

The formation mechanism of high-pressure injection deficiency in low-permeability reservoirs arises from the inability of injected water to overcome the capillary front during waterflooding, leading to diminished sweep efficiency. Over time, ionic incompatibility between formation water (rich in Ca^2+^) and injection water (high in SO_4_^2—^) triggers the nucleation of CaSO_4_ nanoparticles, which evolve into colloidal aggregates under reservoir conditions. These colloids undergo Ostwald ripening and coalescence, ultimately forming crystalline precipitates that occlude pore throats. This dual process—colloid-induced sweep reduction and precipitate-driven pore blockage—serves as the primary driver of elevated displacement pressure in chemically heterogeneous systems.

During the waterflood process in the low-permeability reservoirs, the main controlling factors leading to high-pressure injection deficiency include two aspects. On one hand, wettability plays a crucial role in the waterflood process. It affects the microscopic distribution of oil and water in rock pores, the magnitude and direction of capillary pressure within throats and pores, thereby determining oil displacement efficiency and recovery rate. On the other hand, poor water compatibility is also one of the important factors leading to high-pressure injection deficiency. Poor compatibility between the two water qualities disrupts the original equilibrium state in the formation. Mixing SO_4_^2—^ from the injection water with Ca^2+^ and Ba^2+^ from the formation water can produce precipitates, affecting pore connectivity. The poor wettability (weakly oil-wet) of the low-permeability reservoirs makes it easier to form clustered remaining oil. Clustered remaining oil can form a static mixing zone, and poor water compatibility can cause pore blockage.

**FIGURE 6:**
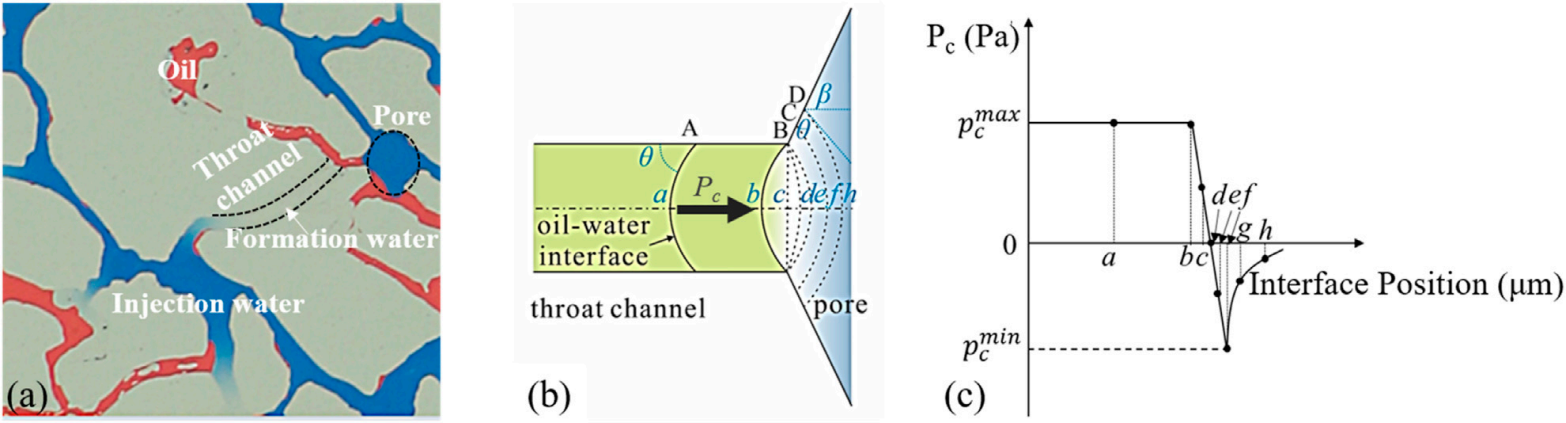
**(a)** Microscopic view of fluid distribution near the pore–throat interface. **(b)** Geometric schematic of capillary valve behavior where capillary pressure reverses at the junction. **(c)** Capillary pressure evolution curve along interface positions a–h, revealing sharp pressure drop indicative of capillary resistance effect.

During the process of waterflooding, capillary force has a significant impact on the distribution of oil and water states. Under the influence of capillary force, in addition to forming film-like and cluster-like remaining oil, the low-permeability reservoirs chip also creates a retention zone within the throat. This retention zone is a stagnant area between the injection water and the oil in the pores, where the injection water cannot contact the oil in the pores, forming a transition zone with the formation water (Figure 6a).

The mechanism for the formation of the retention zone is primarily influenced by capillary force, which causes the oil-water interface to stagnate at the intersection of the throat and pore, impeding oil and water migration. Under water-wet conditions, the direction of capillary force is opposite in the throat and pores (Figure 6b). When the oil-water interface is in the throat, the direction of capillary force (pointing to the concave side of the liquid surface) aligns with the direction of oil-water movement under water-wet conditions, making capillary force a driving force (A→B). However, when water exits the throat and θ + β > π/2, the oil-water interface reverses and eventually stops moving due to capillary resistance. This phenomenon is further quantified by the capillary pressure profile shown in Figure 6c, which illustrates a sharp pressure drop across the interface positions and confirms the presence of a capillary barrier. Therefore, the driving pressure must be higher than the negative maximum value, $p_{c}^{\min}$ , to facilitate fluid movement. This threshold is known as the capillary valve, and the corresponding pressure condition is expressed in Equation 1.

$$p_{c}^{\min}=\frac{\sigma \cos\left(\theta +\beta \right)}{r}$$ (1)

where $p_{c}^{\min}$ is the interfacial tension coefficient, *θ* is the wetting angle, *β* is the pore’s opening angle, and *r* is the equivalent radius of the channel.

### 3.3 Waterflooding with formation water

**FIGURE 7:**
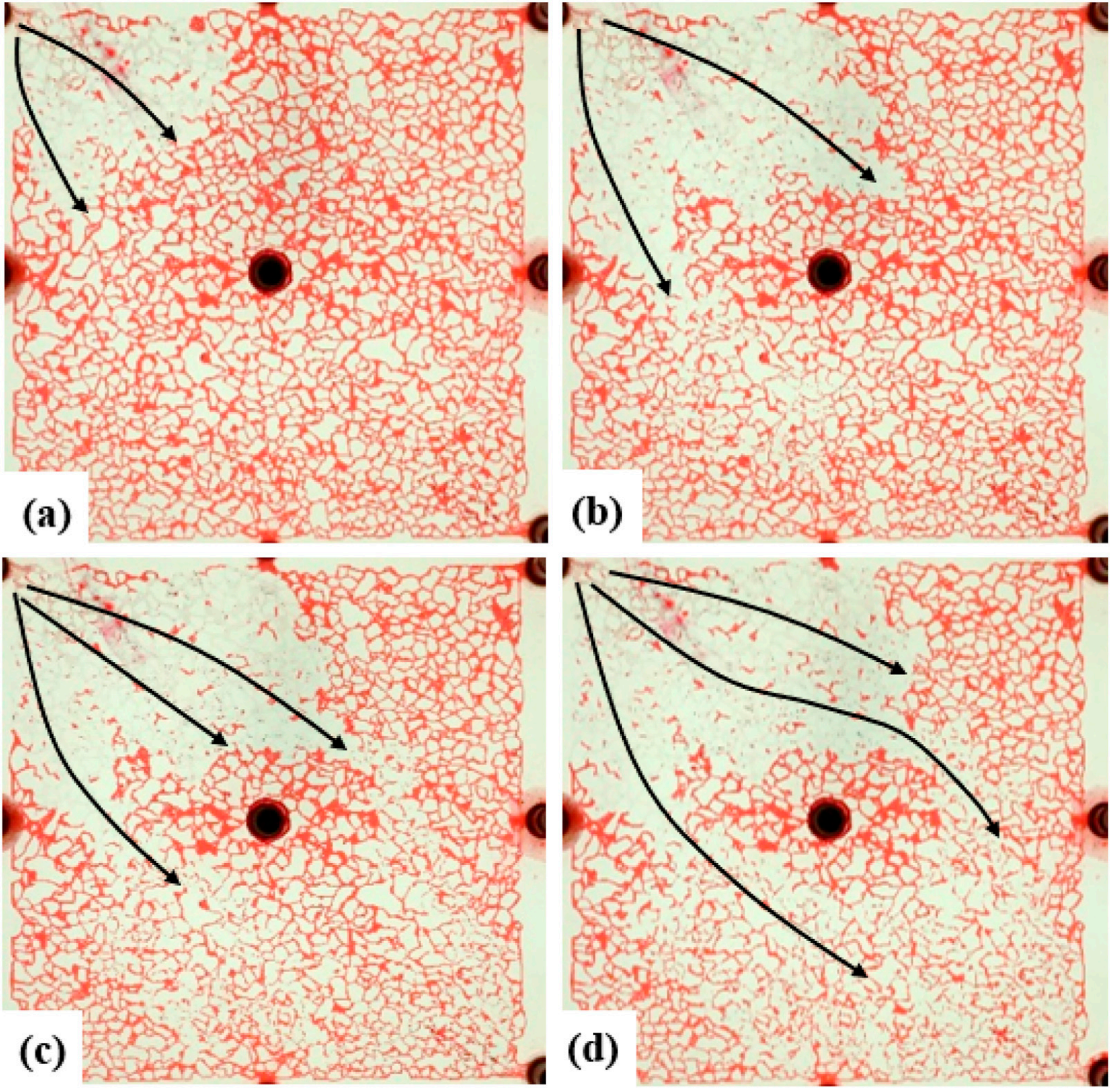
Microscopic images of oil–water distribution at different injection pore volumes: **(a)** 0.1 PV, **(b)** 0.3 PV, **(c)** 0.5 PV, and **(d)** 0.7 PV. Red and white represent oil and formation water, respectively. The displacement front advances progressively with increasing injection volume.

**FIGURE 8:**
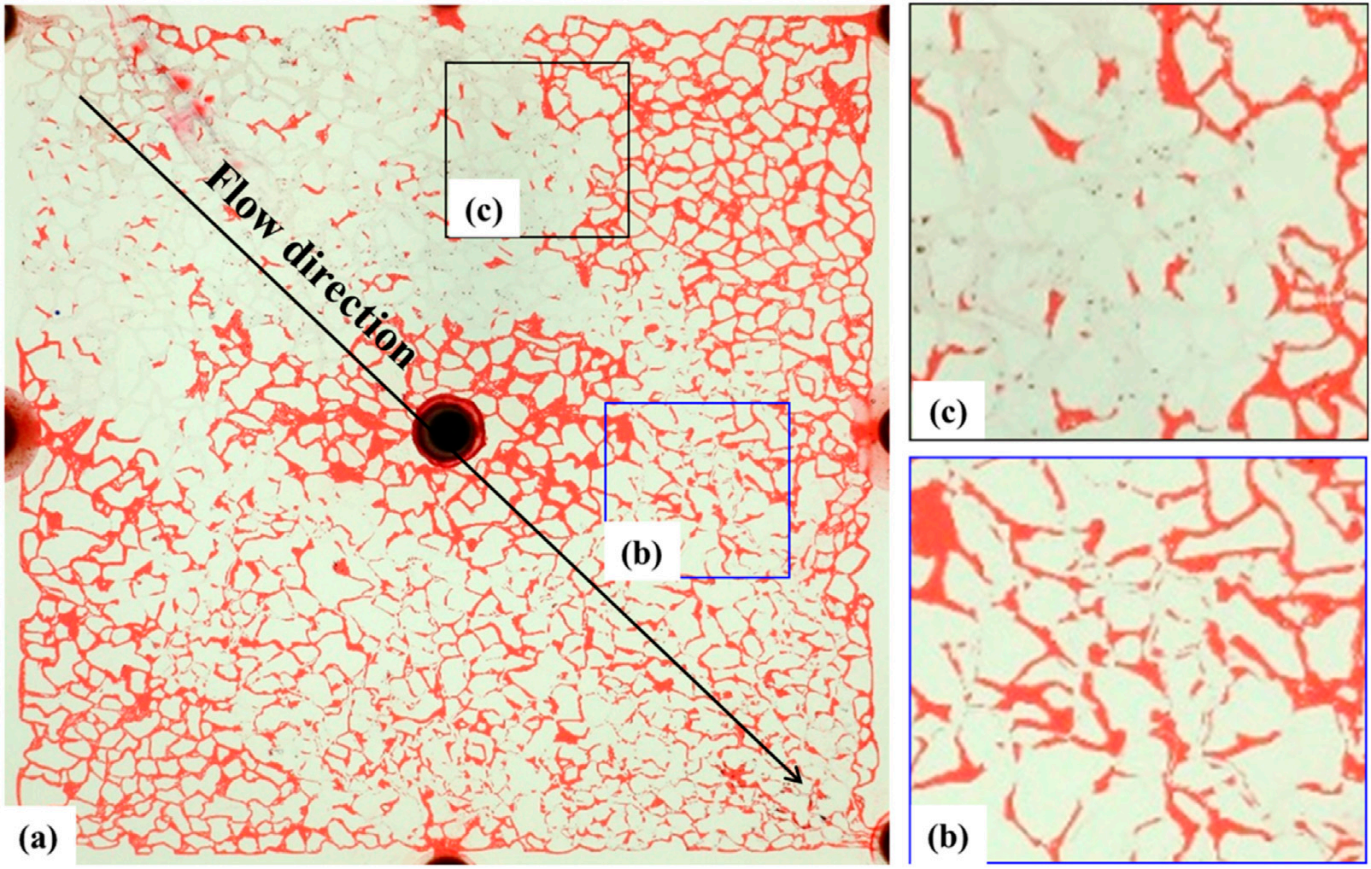
Final oil–water distribution after primary water flooding in a low-permeability reservoir. **(a)** Overall microscopic view; **(b)** Remaining oil in a film-like distribution; **(c)** Remaining oil in a cluster-like distribution. Color scheme is consistent with Figure 7.

The water flooding speed in the low-permeability reservoirs was set at 2 μL/min. After the water flooding experiment began, water continuously broke through from the inlet, ultimately forming two water channeling paths (Figure 7). When water flooding reached a high water content stage, there was still a large area of remaining oil on both sides of the main flow channel. Due to the relatively small fluidity ratio of oil and water, there was relatively little remaining oil in the main flow channel, mainly consisting of film-like and cluster-like remaining oil (Figure 8).

The high-resolution image (Figure 8) further indicates: Apart from the unreached areas on both sides of the mainstream zone where the remaining oil completely filled the pores, the remaining oil in the center of the mainstream zone was mainly film-like. Due to the high salinity of the formation water and the interfacial tension between oil and water, an oil-wet wall condition was formed after water flooding. Additionally, there were small amounts of cluster-like remaining oil, oil droplets, and pinch-offs in the pores. The former occupied pores in large, continuous blocks, while the latter appeared to be trapped in the pores. Because the oil reservoir chip had water-wet conditions, during the water flooding process, the water film advanced along the wall at some locations, causing the remaining oil to be located in the center of the pores or attached to one side of the wall.

### 3.4 Waterflooding with injection water

**FIGURE 9:**
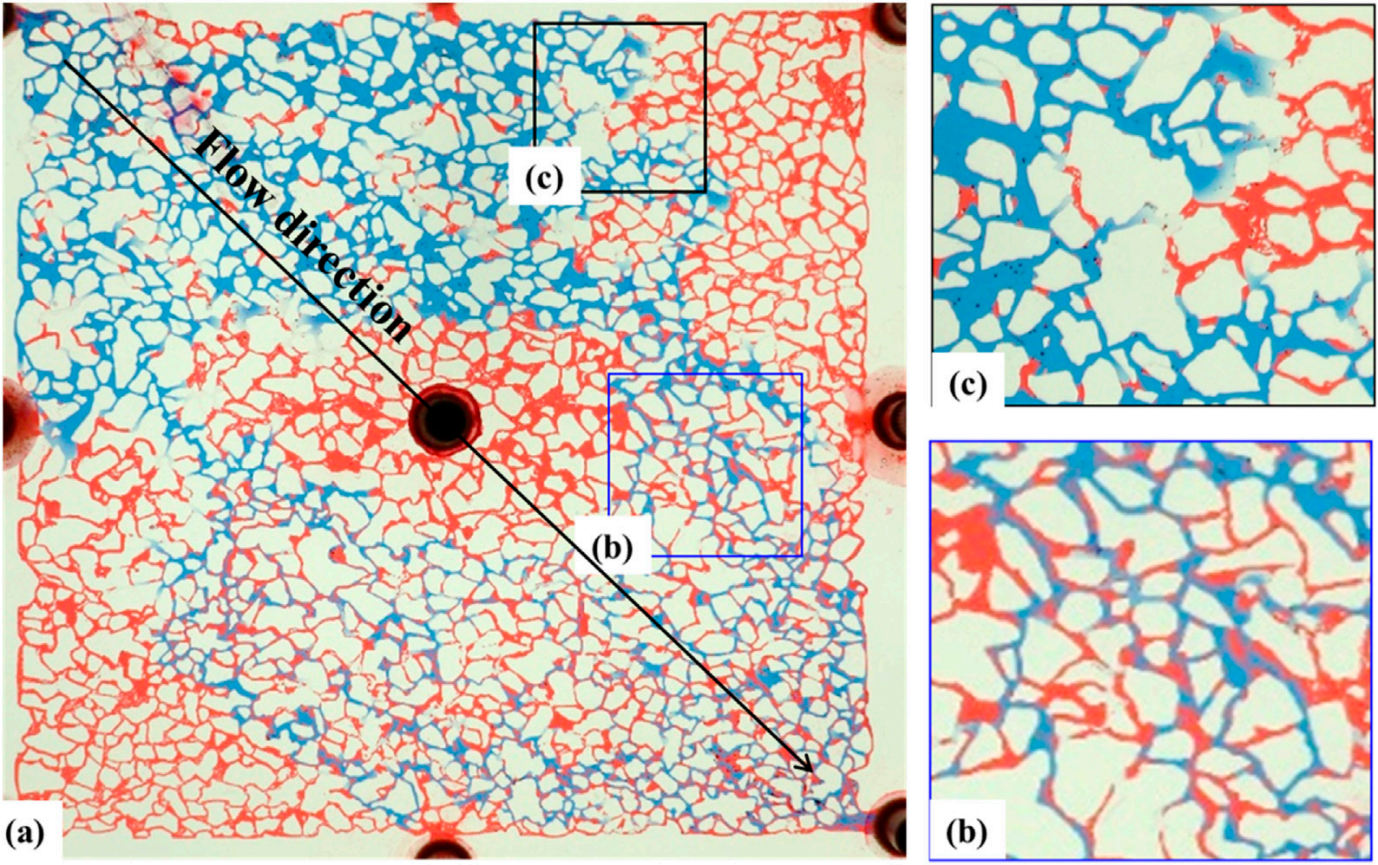
Microscopic oil–water distribution after secondary flooding with injection water. **(a)** Final distribution of residual oil following sequential injection of formation and incompatible water; **(b)** Film-like residual oil adhering to pore walls; **(c)** Cluster-like residual oil trapped in pore centers. Red, blue, and white represent oil, injection water, and formation water, respectively.

After the formation water flooding is over, the water flooding process is continued with Injected water. The water injection rate was maintained at 2 μL/min, and the distribution of remaining oil after waterflooding is shown in Figure 9. The injection water had a wider sweep range than the formation water, primarily displacing film-like and clustered oil while expanding the boundaries of the waterflood. The reduction in film-like and clustered remaining oil was attributed to the inherently water-wet conditions of the reservoir. The high salinity of the formation water (9.3 × 10^4^ mg/L) resulted in a weaker water-wetting effect on the wall surface, whereas the lower salinity of the injection water (0.2 × 10^4^ mg/L) exhibited a stronger water-wetting effect.

Simultaneously, it was observed that the injection water drove the formation water towards the remaining oil in the marginal seepage channels. However, the two types of water were not completely miscible, resulting in a transition zone between them (Figure 9c). The injection water could not break through the front of the formation water, indicating a physical boundary between the two waters. This incomplete miscibility suggests a compatibility mismatch between the injection water and the formation water. Additionally, the incomplete miscibility and the delay in wall wettability changing to water-wet contributed to the rise in displacement pressure.

### 3.5 Surfactant suppress scale deposition

After the initial water flooding with injection water, a continued injection was carried out at a rate of 2 μL/min. Once no oil was observed at the outlet, a solution of 3% COA-2G and 0.5% NH_4_Cl prepared with injection water was injected. The pore-scale flow behaviors of oil and water during this process, as well as the pressure variations at the injection inlet, were analyzed.

**FIGURE 10:**
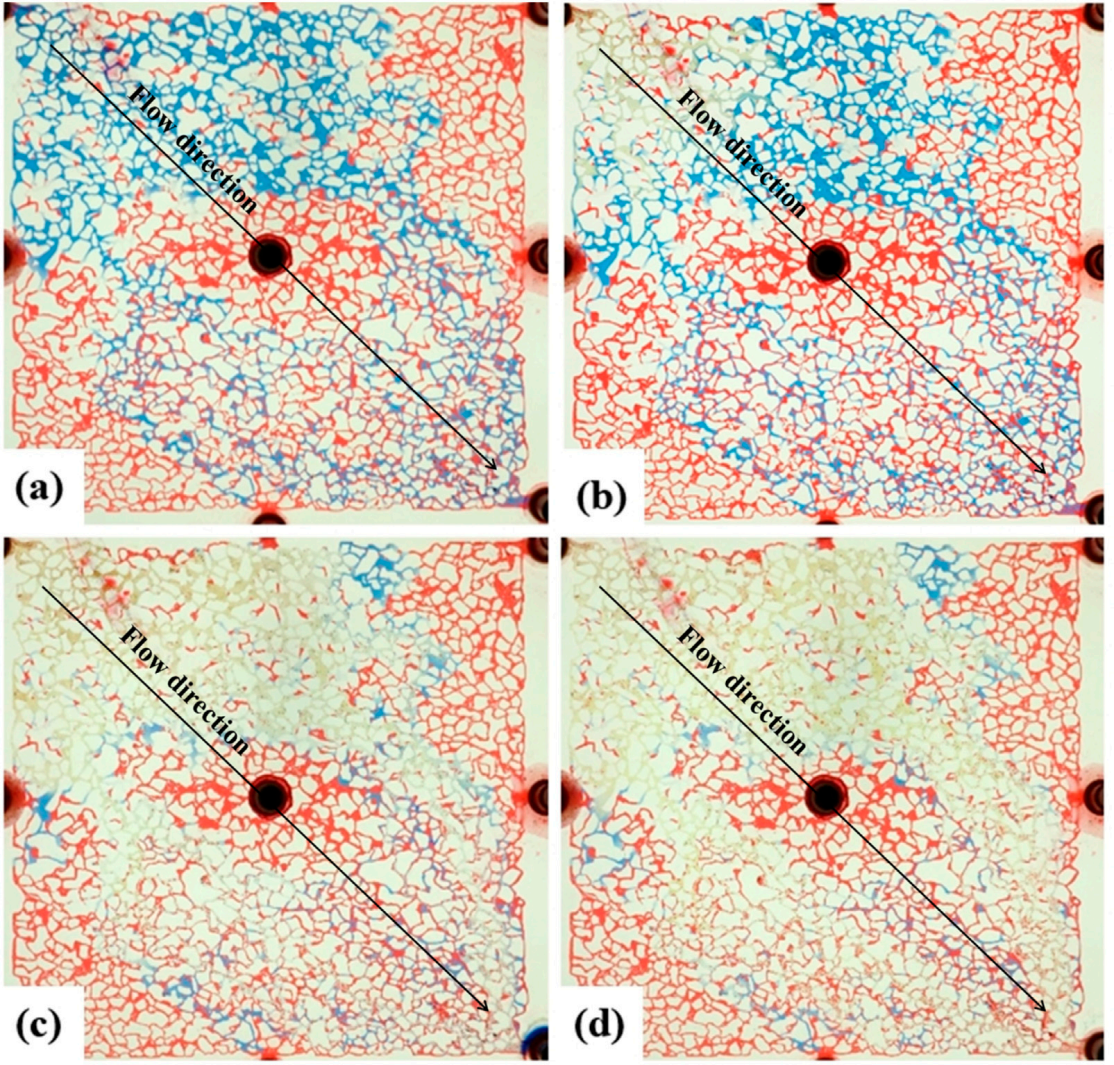
Microscopic visualization of oil displacement during surfactant flooding at different injection volumes: **(a)** 0.1 PV, **(b)** 0.3 PV, **(c)** 0.5 PV, and **(d)** 0.7 PV. Progressive advancement of the displacement front and reduction in residual oil saturation are observed. Red, blue, white, and yellow represent oil, injection water, formation water, and the chemical flooding solution (surfactant and ammonium chloride), respectively.

As shown in Figures 10a–d, the surfactant solution initially displaced the oil in the channels formed during the primary injection water flooding. In Figures 10a, the surfactant solution begins to penetrate, targeting the high-permeability regions of the core model. As the injection progresses to Figures 10b, the surfactant solution moves deeper into the pore network, gradually mobilizing residual oil trapped in bypassed areas. This process is particularly effective in medium-permeability regions, where oil droplets are emulsified and begin to migrate with the surfactant flow. In Figures 10c, the surfactant solution covers a wider sweep area, reaching previously inaccessible oil in low-permeability regions. The emulsification effect becomes more pronounced, and the oil is further mobilized, with the displacement front moving efficiently across the core. As shown in Figures 10d, the surfactant solution has fully displaced the injection water and reached the outlet, with no further oil production. The surfactant has completely occupied the flow paths of the injection water, and the sweep area continues to expand, displacing more oil from the surrounding pores.

**FIGURE 11:**
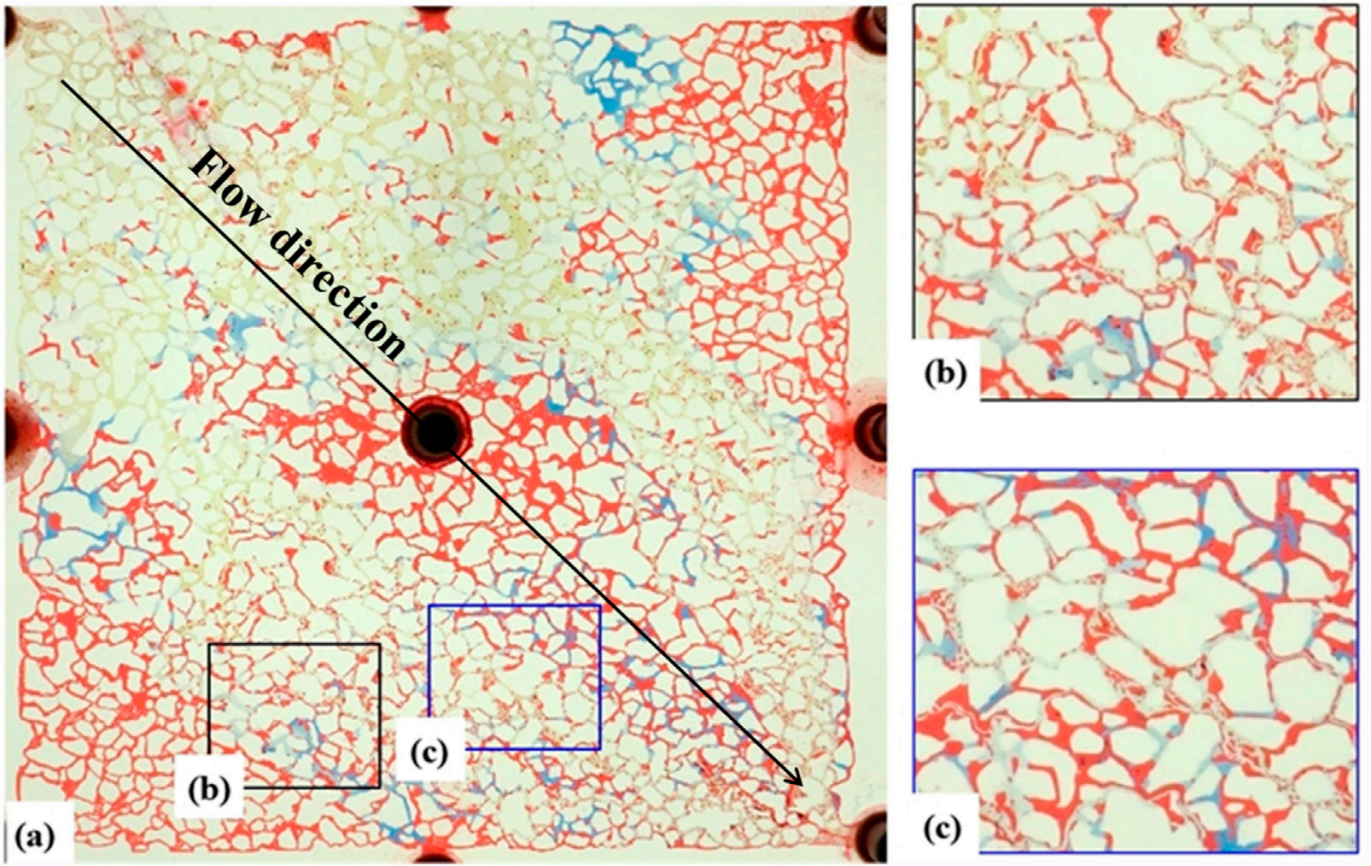
Emulsification behavior during surfactant flooding. **(a)** Overall microscopic view of the porous medium; **(b,c)** Magnified views showing dispersed oil droplets suspended within the chemical flooding phase. Red, blue, white, and yellow represent oil, injection water, formation water, and the surfactant–ammonium chloride solution, respectively.

Figure 11 illustrates the emulsification phenomenon in the main flow channels. The interaction between the surfactant and the oil leads to a significant reduction in oil viscosity and the transformation of the oil phase into emulsified droplets. This process is crucial for improving the mobility of trapped oil. As the oil-water interfacial tension decreases, oil droplets break up and disperse, facilitating their movement toward the outlet. Additionally, the wettability of the pore surfaces changes from oil-wet to water-wet, further enhancing oil recovery. This interfacial alteration enables more oil to become mobile and, ultimately, be displaced by the surfactant solution, increasing the overall displacement efficiency. The emulsification and wettability alteration observed in Figure 11 highlight the surfactant’s critical role in improving microscopic sweep efficiency. By converting oil-wet surfaces to water-wet, the surfactant facilitates the release of previously trapped oil, ensuring a higher displacement efficiency compared to traditional water flooding. This enhanced oil recovery mechanism significantly contributes to the success of the surfactant-driven displacement process.

However, the mixing of injection water and formation water leads to the formation of colloids or precipitates, causing difficulties for the surfactant’s movement at the oil-water interface. This results in the clustering of oil droplets and reduces the effectiveness of oil mobilization through interfacial tension reduction. To address this issue, 0.5% NH_4_Cl was added to the COA-2G surfactant solution to promote the dissolution of CaSO_4_ and slightly increase its solubility. Given that the concentration product of Ca^2+^ in formation water and SO_4_^2-^ in injection water is 70.5–189.9 times higher than the solubility product of CaSO_4_, the addition of a small amount of NH_4_Cl cannot fundamentally resolve the problem of CaSO_4_ precipitation.

**FIGURE 12:**
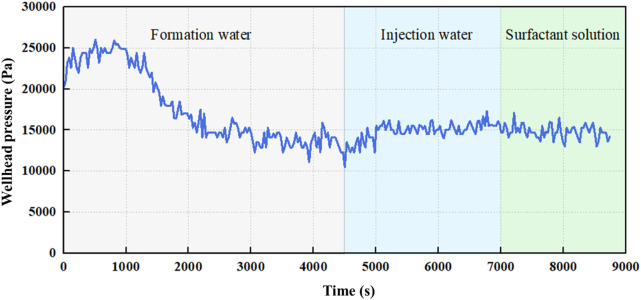
Wellhead pressure response during sequential flooding with formation water, injection water, and surfactant solution. The timeline is divided into three shaded regions indicating the three injection stages. Blue curve denotes dynamic pressure at the inlet. Gray, blue, and green zones correspond to formation water injection, incompatible injection water flooding, and chemical flooding with surfactant solution, respectively.

Figure 12 presents the real-time wellhead pressure response over the full course of the displacement experiment, segmented into three distinct fluid injection stages: formation water, injection water, and surfactant solution. During the formation water injection stage (0–4500 s), the injection pressure rises from approximately 22,000 Pa to a peak of nearly 26,000 Pa, indicating increased displacement resistance due to advancing oil fronts and capillary trapping. As breakthrough occurs, the pressure drops sharply and stabilizes around 14,500 Pa, suggesting establishment of a two-phase flow regime. In the subsequent injection water stage (4500–7,000 s), where the injected brine is chemically incompatible with the formation water, the pressure rises slightly to an average level of 15,500 Pa. This secondary increase reflects the onset of scale precipitation (e.g., CaSO_4_, BaSO_4_), leading to additional flow resistance and localized pore blockage. Finally, during surfactant flooding (7,000–9,000 s), the pressure exhibits a mild decline, stabilizing around 13,500 Pa. This reduction results from surfactant-induced interfacial tension lowering, improved wettability, and possible partial re-opening of clogged pores. The system reaches a new quasi-steady state with enhanced oil mobility and reduced hydraulic resistance. This pressure evolution provides compelling evidence of the dynamic coupling between chemical incompatibility, scale formation, and chemical remediation, underscoring the importance of tailored injection strategies for complex reservoirs.

## 4 Conclusion

This study systematically investigated the pore-scale mechanisms of chemical incompatibility between formation and injection water and its implications for EOR in low-permeability reservoirs. By integrating ion characterization, colloidal dynamics analysis, microfluidic visualization, and pressure monitoring, we elucidated the interplay between scaling deposition, fluid trapping, and surfactant-mediated mitigation. The key findings are summarized as follows:

1. Chemical incompatibility drives pore-throat blockage through a dual-stage mechanism: (i) rapid nucleation of CaSO_4_/BaSO_4_ nanoparticles due to ionic interactions between sulfate-rich injection water and divalent-cation-rich formation water, and (ii) progressive growth of crystalline precipitates via coalescence or aggregation. These processes obstruct pore throats, reducing sweep efficiency and significantly elevating injection pressure.
2. Capillary valve effects intensify fluid trapping in stagnant zones, where residual oil persists as interfacial films and pore-center clusters. Incompatible water injection amplifies trapping by forming immiscible transition zones, further hindering oil mobilization. Wettability alteration under low-salinity conditions partially mitigates this effect but cannot fully counteract scaling-induced permeability impairment.
3. Synergistic surfactant-scale inhibitor formulations enhance recovery by simultaneously reducing interfacial tension and suppressing scale nucleation. The combined chemical strategy improved emulsification efficiency and mobilized additional residual oil compared to conventional waterflooding, though solubility limitations of scaling species necessitate further optimization.
4. A colloid-capillary coupling framework is proposed to describe the self-reinforcing cycle of scaling (nanoparticle generation → capillary trapping → precipitate growth). Disrupting this cycle requires dual interventions: wettability modification to reduce capillary resistance and nucleation inhibition to delay colloidal aggregation.
5. Microfluidic validation highlighted preferential scaling at throat-pore junctions, where stagnant zones promote particle accumulation. Surfactant flooding expanded sweep efficiency but faced limitations in low-permeability regions due to persistent scale deposits, emphasizing the need for proactive compatibility management.

The insights gained from this study have practical implications for the design of waterflooding operations in chemically sensitive, low-permeability reservoirs. By directly visualizing pore-scale particle accumulation and blockage formation under ion-incompatible injection, the results highlight the need for precise control over injection water chemistry to prevent permeability impairment. The identification of capillary valve effects and dynamic scaling behavior also underscores the value of real-time flow monitoring during field-scale water injection. Moving forward, this experimental approach can be extended to explore more complex chemical systems involving multivalent ions, organic additives, or fluctuating temperature and salinity conditions, providing a foundation for tailored mitigation strategies in heterogeneous reservoir environments. These findings inform injection strategy design for chemically heterogeneous reservoirs and guide real-time compatibility monitoring during waterflooding operations.

## Data Availability

The original contributions presented in the study are included in the article/supplementary material, further inquiries can be directed to the corresponding authors.
